# Development of real-time PCR for detection of *Mycoplasma hominis*

**DOI:** 10.1186/1471-2180-4-35

**Published:** 2004-09-06

**Authors:** Agata Baczynska, Helle F Svenstrup, Jens Fedder, Svend Birkelund, Gunna Christiansen

**Affiliations:** 1 Department of Medical Microbiology and Immunology, Aarhus University, The Bartholin Building, 8000 Aarhus C, Denmark; 2 The Fertility Clinic, Brædstrup Hospital, 8740 Brædstrup, Denmark

**Keywords:** Fluorescence probes, *gap gene*, LightCycler PCR,*Mycoplasma hominis*, real-time PCR

## Abstract

**Background:**

*Mycoplasma hominis *is associated with pelvic inflammatory disease, bacterial vaginosis, post partum fever, sepsis and infections of the central nervous system often leading to serious conditions. Association with development of female infertility has also been suggested, but different publications present different results. We developed a sensitive and fast diagnostic real-time PCR to test clinical samples from women undergoing laparoscopic examination before fertility treatment. To develop a test for the detection and quantification of *M. hominis *we selected a housekeeping gene, glyceraldehyde-3-phosphate dehydrogenase (*gap*), as a target.

**Results:**

Real-time PCR was optimized to detect 10 copies of *M. hominis *PG21 genomic DNA. A fluorescence signal was measured for all 20 other *M. hominis *isolates, and melting curves analysis showed variations in the melting temperature in agreement with sequence variation in the region of the probes. There was no amplification of other mycoplasmal DNA and human DNA. Eighty-three patient cervical swab samples from infertile women were cultured for *M. hominis *in the BEa medium. Two of the samples (2.4%) were positive after 48 hours of incubation. The real-time PCR detected the same two samples positive, and the DNA concentrations in the clinical specimens were calculated to 37.000 copies/ml and 88.500 copies/ml, respectively.

**Conclusion:**

The results demonstrate that real-time PCR may prove to be a rapid alternative to the traditional cultivation method. Information on bacterial load in genital swabs can be obtained. The assay allowed detection of *M. hominis *in a closed system reducing the risk of contamination by amplicon carry-over.

## Background

Mycoplasmas are the smallest living prokaryotes known, capable of self-replication. They belong to the class *Mollicutes *and are distinguished phenotypically from other bacteria by their minute size and lack of a cell wall. Genetically they differ by having a small genome size and low G+C content {1} [1]. Mycoplasmas have adapted to a wide variety of hosts and can colonize man, other animals and plants. The colonising organisms are host specific. In humans, mycoplasmas colonize mainly the upper respiratory tract and the genitourinary tract.

The first human *Mycoplasma *isolated was *Mycoplasma hominis *[2]. It is a heterogeneous genital mycoplasma [3] found in at least two-thirds of women with bacterial vaginosis (BV), compared to 10% of healthy women [4,5]. *M. hominis *has also been isolated from the endometrium and fallopian tubes of 10% women with salpingitis. However, its role as a primary pathogen is doubtful since it co-exists with many other bacteria in BV [6]. Studies made on women undergoing *in vitro *fertilization showed the presence of *M. hominis *only in 2.1% of the women [7].

Isolation from other sites than the genitourinary tract has been reported. *M. hominis *has been found to cause wound, joint and central nervous system infections [8], it has been isolated from cavernous angioma, but was not the cause of the disease [9] and cases of brain and scalp abscesses and meningoencephalitis were also reported [10-15]. Those cases demonstrated the pathogenic potentials of *M. hominis *and indicated a need for rapid recognition. So far culture is most commonly used for detection of genital *Mycoplasma, *but it requires special handling, complex media, and cultivation positive samples need further testing to determinate the species cultivated [16]. A case report of brain abscess in a 22 year-old female patient with postpartum infection [10] showed that culture took 4 – 5 days during which the patient's symptoms continued to worsen before the antibiotic treatment was changed.

Comparison between culture method and PCR has been performed and showed that a PCR assay was as sensitive as culture for detection of *M. hominis *from clinical samples. In addition it was very specific [17]. An advantage of using PCR is that the system can detect the presence of both live and dead microorganisms in the sample. When comparing the original PCR protocols with the newly developed real-time protocol, it offers interesting advantages such as rapidity, closed system, which eliminates the risk of carry-overs, real-time monitoring of PCR activity, quantification of amplification product and, if required, mutation analysis.

A recent study designed for the detection of *M. hominis *by real-time PCR in HIV-positive patient swab samples, suggested the use of SYBR Green with primers targeting another housekeeping gene, the 16S rRNA gene [18]. The 16S rRNA gene is the most conserved microbial gene, though, in *M. hominis *minor sequence variation was observed [19]. Because sequences from *M. hominis *isolates were available (see table 1 for accession nos.) and possible to compare with other mycoplasmas sequence [28-30], we selected the housekeeping gene glyceraldehyde-3-phosphate dehydrogenase (*gap*) as target for development of a quantitative real-time PCR. Comparison of the DNA sequences from different *M. hominis *isolates showed, however, some small variations in the amplified DNA sequence [31]. We determined sensitivity and specificity of the *M. hominis *LightCycler real-time PCR and tested it on clinical swab samples using specific hybridization probes for detection.

## Results

### Design of primers and probes

The principles of the system we used are based on two specific hybridization probes located internally to the amplification primers, each of them labelled with a different fluorescent dye [32]. The DNA sequence of primers and probes is shown (table 2).

**Figure 1:**
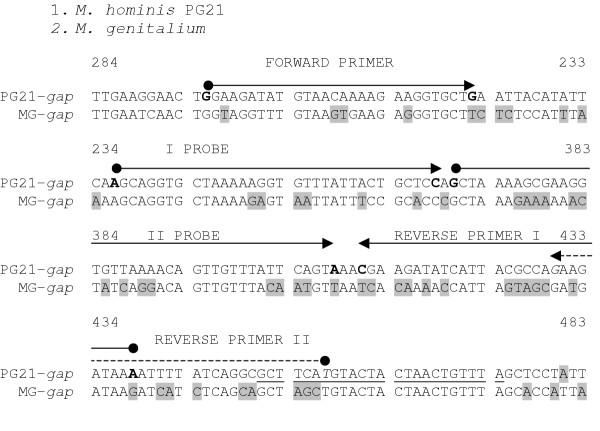
**Orientation of primers and probes with sequence comparison to the closer related genital bacteria *M. genitalium. ***The DNA sequence of primers and probes from *M hominis *PG21 is compared to the homologous *M. genitalium *sequence. Eight mismatches were present in the forward primer, 7 in the first probe, 15 in the second probe and 13 in the reverse primer I. Highlighted nucleotides correspond to mismatches. Letters in bold correspond to the beginnings and ends of designed primers (forward and reverse I) and probes (I, II). Letters in italic correspond to beginning and ending of reverse primer II. The active site region of GAPDH enzyme is underlined.

We selected *M. hominis *PG21 type strain as a template for primers and probes. The primers and probes were designed with respect to conservation of the DNA sequence within the *M. hominis gap *gene [31] and difference from the related genital *Mycoplasma genitalium gap *gene (Accession no. U39710) (fig. 1). The amplified DNA fragment was of 144 bp in size.

Swissprot Protein Database was used to determine the amino acids sequence of GAPDH enzyme (E.C. no. 1.2.1.12). By use of the MOTIFS program the active site was predicted to consist of the amino acids: ASCTTNCL, located at nucleotides 451 to 474 (fig. 1) [31]. When comparing DNA sequences of the active site between *M. hominis *and *M. genitalium, *only 3 out of 24 nucleotides are mismatching (fig. 1), and therefore the probes and primers were placed before this region.

As seen from fig. 1, the G + C content of the *M. hominis gap *gene is very low. It was difficult to find a suitable location for the reverse primer, and therefore two reverse primers were designed (table 2). The sequence of the reverse primer II was partly overlapping the encoding region of the active site of GAPDH enzyme. When compared it was found that use of reverse primer I gave the highest sensitivity of the LightCycler PCR assay. Concentration of 5 copies/μl of PG21 DNA was not always detected in PCR runs when reverse primer II was used, whereas with use of reverse primer I, 5 copies/μl of PG21 were present in every run, and therefore this primer was chosen for the following experiments.

In order to obtain optimal detection in the annealing phase of the PCR we designed the probes to anneal to the same strand as the forward primer and placed them as far as possible from that primer (fig. 1). One hybridization probe was labelled with fluorescein (FL) in the 3' end, the other with LightCycler Red705 (LCred705) in the 5' end. When the probes are hybridized less than 5 nucleotides apart, Fluorescence Resonance Energy Transfer (FRET) will be induced. The distance between the two probes was one nucleotide after annealing to template DNA allowing FRET light to be measured.

### Sensitivity and specificity

**Figure 2:**
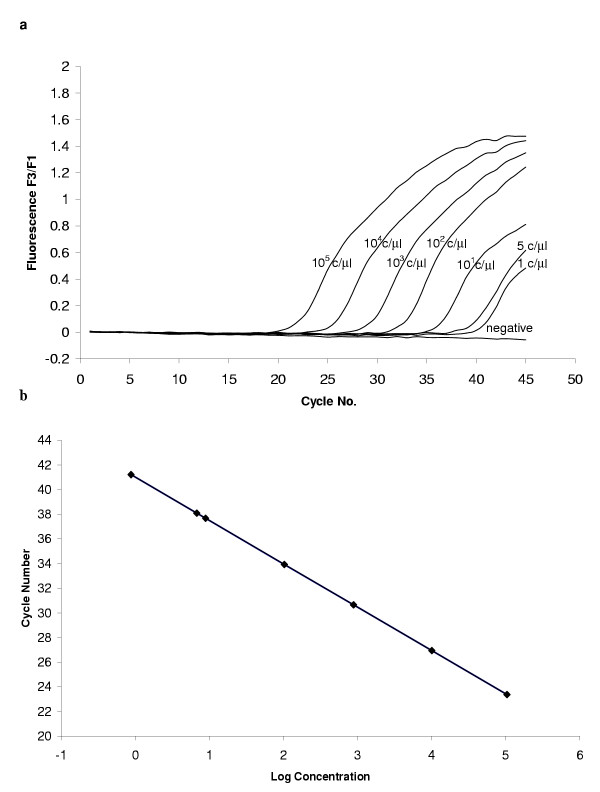
**Sensitivity of LightCycler PCR in standard dilution series. **(a) The LightCycler PCR run with *M. hominis *PG21 DNA and fluorescence probes was done. The fluorescence signal of 10-fold dilution series from 10^5 ^to 5 and 1 copy is shown. No reaction was noted in the negative control (0 copies). (b) Standard curve was generated from the threshold cycles (*C*_*t*_) also known as crossing points (*Cp*) of the *M. hominis *PG21 standard dilution series by the LightCycler software.

Dilution series of the standard DNA from *M. hominis *PG21 were used to examine the sensitivity of the LightCycler PCR assay. The fluorescence curves are shown (fig. 2a). Detection of PCR product was possible for the lowest DNA concentration: 1 copy/μl, equal to 2 copies in the reaction mixture (fig. 2a). To reduce the noise, the cut-off value was set to 0.1. The standard curve had an average slope equal to -3.5, which means that the efficiency of the PCR reaction was 1.93 (oscillating between 1.9 and 2.0) (fig. 2b). Calculations of the efficiency derived from the function for the amount of PCR product that was formed, represented by equation: N = N_0 _× 2^n^, where N is the number of amplified product, N_0 _is the initial number of molecules and n is the number of the PCR cycles. Ideally the efficiency equals 2. Additionally, the sensitivity of the assay was determined for two other *M. hominis *isolates by making dilution series of DNA from *M. hominis *132 and 4712. As for PG21, detection of a PCR product was possible for the lowest DNA concentration of 1 copy/μl indicating similar sensitivity of the two other isolates. The standard curves had slopes equal to -3.552 for isolate 132 and -3.576 for isolate 4712, the efficiency of the PCR reaction was 1.91 and 1.9, respectively.

To determine the reproducibility of the assay, ten dilution series of PG21 DNA were analyzed in different PCR runs, and the values of crossing-points (also known as threshold cycles – *C*_*t*_) were compared by calculation of the coefficients of variation (*CV*). The values of *CV *were between 3.8% and 6.7% (table 3). In all runs 10 out of 10 samples were positive except for 1 copy/μl where a PCR product was seen in 6 out of 10 PCR runs. The reproducibility of the assay for the two other *M. hominis *isolates 132 and 4712 was analyzed in three different PCR runs. In both isolates dilution of 5 copy/μl was present in three out of three runs, similarly to PG21 the dilution of 1 copy/μl was present in two out of three LightCycler runs. Our detection limit was therefore 5 copy/μl, equals to 10 copies in the reaction mixture. The reproducibility of the assay was acceptable.

**Figure 3:**
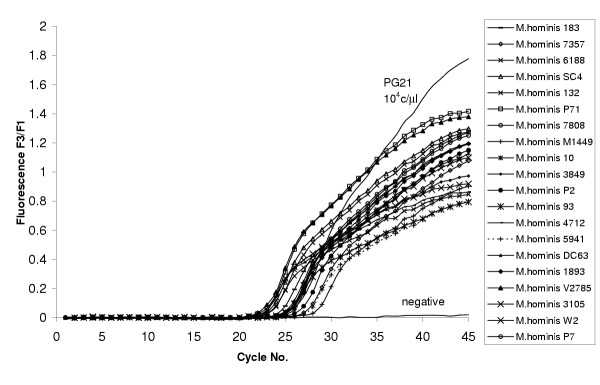
**Specificity of the LightCycler PCR. **The LightCycler PCR run with different *M. hominis *isolates. Concentration of DNA from different isolates used in the study was estimated to 10^4 ^copies/μl of PG21 DNA, which was used as a standard DNA.

As a next step twenty *M. hominis *isolates (table 1) were tested with the designed primers and probes in the LightCycler real-time PCR. All isolates gave a positive fluorescence signal and the concentration of DNA was similar to 10^4 ^copies/μl of PG21, measured by the LightCycler instrument (fig. 3).

**Figure 4:**
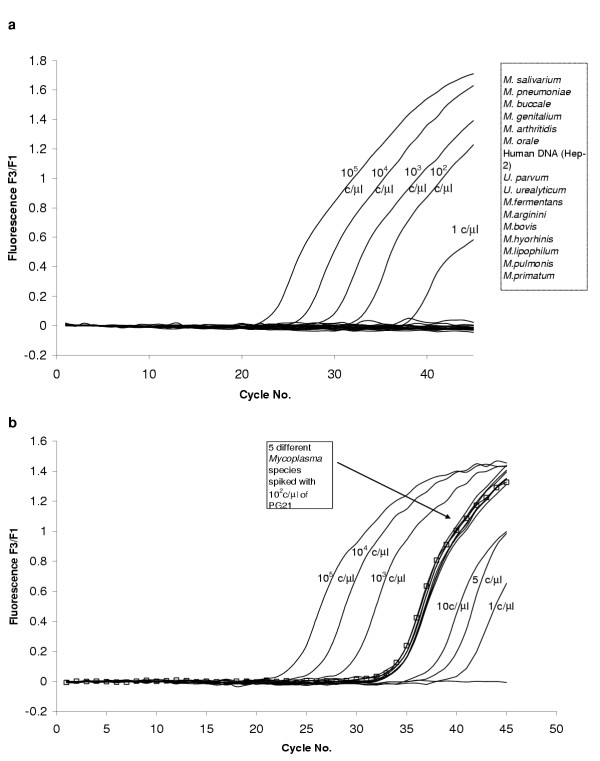
**Specificty of the LightCycler PCR. **(a) LightCycler PCR with human DNA from Hep2 cells and selected *Mycoplasma spp. *run with the designed primers and probes. (b) Spiking assay showed no inhibition when DNA from five different *Mycoplasma *species (*M. arginini, M. bovis, M. hyorhinis, M. pulmonis, M. salivarium) *was spiked with PG21 DNA of 10^2 ^copies/μl. All curves came up at the same time as 10^2 ^copies/μl of PG21 from the standard dilution series (marked with squares).

The specificity of the LightCycler assay targeting the *gap *gene was evaluated by testing human DNA and DNA from different *Mycoplasma *species. With the specific probes there was no cross-reactivity to other *Mycoplasma *species or human DNA (fig. 4a). For human DNA we used a concentration of 10^4 ^copies/μl calculated by OD measurements of the purified DNA and the genome size.

Since DNA from different mycoplasmas was extracted by proteinase K treatment of PBS washed pellets, we tested such extracted DNA for presence of inhibitors. Two μl of DNA from five different mycoplasmas (*M. arginini, M. bovis, M. hyorhinis, M. pulmonis, M. salivarium*) were spiked with 2 μl of PG21 DNA of 100 copies/μl and added to the reaction mix. As illustrated (fig. 4b) there was no inhibition in the proteinase K treated samples.

### Melting curve analysis

**Figure 5:**
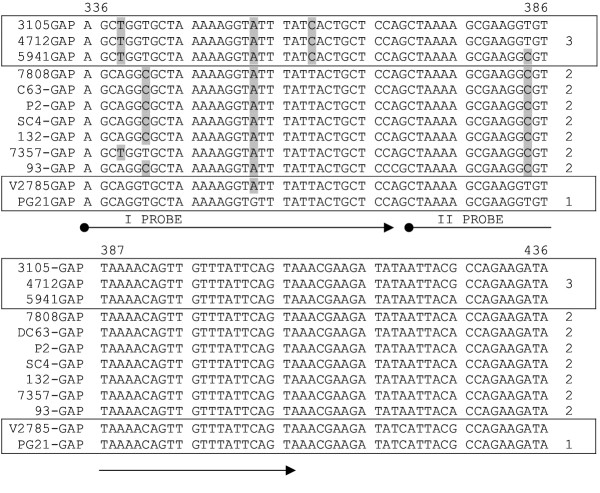
**Sequence alignment of the probe region of *M. hominis *isolates. **The sequence of PG21 was used as a template. The probe sequences from the *M. hominis *isolates are shown. Highlighted base pairs corresponded to differences in probe sequences in comparison to PG21 standard. Groups are shown in boxes: top box represents the lowest melting temperature of 62°C, group 3, with isolates; 3105, 4712 and 5941; medium box contains: 7808, DC63, P2, SC4, 132, 7357 and 93 that have a melting temperature of 64°C; and bottom box with V2785 is almost identical to PG21 with only one mismatch and a melting temperature of 66°C.

Melting curve analysis can be used to determine the presence of non-specific amplification products. The melting temperature (*Tm*) is defined as the temperature at which half of a duplex-DNA becomes single-stranded [33]. As it was impossible to place probes in completely conserved regions (fig. 5), we analyzed the melting curves of the real-time PCR products of the different *M. hominis *isolates. *Tm *of PG21 DNA was 66°C and equal for high and low concentrations of DNA.

**Figure 6:**
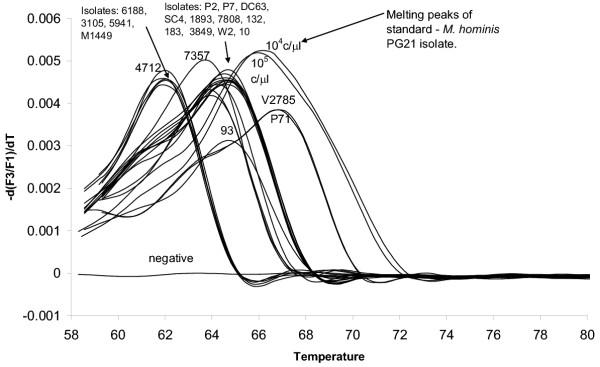
**Melting peak analysis with *M. hominis *isolates. **Melting curve analysis of *M. hominis *isolates was performed after quantification step. The three melting temperatures are marked with arrows. Two concentrations of PG21 DNA standard dilution series are shown. All samples were run with concentration of 10^4 ^copies/μl.

It was shown that the melting temperature of the PCR products of *M. hominis *DNA clustered in 3 major groups (fig. 6). The isolates V2785 and P71 had the same temperature of 66°C as PG21, the second group 93, 7357, 132, P2, P7, SC4, DC63, 7808, 183, 1893, 10, W2 had a melting temperature of 64°C, while the last group 5941, 4712, 3105, M1449, 6188, had the melting temperature of 62°C. These different melting points were in agreement with variation in the DNA sequence of the probe regions (fig. 5).

### Coloured media and possible PCR inhibition

**Figure 7:**
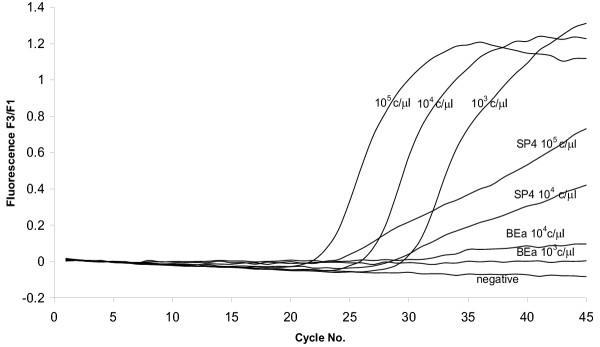
**Inhibition of the BEa and SP-4 media on LightCycler PCR. **LightCycler PCR with SP-4 medium and BEa spiked with PG21 DNA of the concentrations: 10^5^, 10^4^, and 10^3 ^copies/μl, respectively.

Many *Mycoplasma *species were cultured in SP-4 or BEa media for specificity of LightCycler analysis. Additionally, the clinical samples were transported in SP-4 medium, which can be used for the recovery of *M. hominis *[34], and BEa medium was used for cultivation of *M. hominis*. Therefore, to see the effect of the coloured media on the LightCycler assay, we constructed artificial samples consisting of SP-4 or BEa medium spiked with DNA of known concentration (10^5^, 10^4^, 10^3^). Two μl of the samples were analyzed by the LightCycler PCR. As shown (fig. 7), BEa medium completely inhibited the reaction, whereas SP-4 inhibited only partially, but markedly reduced the PCR efficiency.

### Analysis of clinical samples

#### Culture

Eighty-three endocervical samples from women attending fertility clinics in Denmark were cultured for the presence of *M. hominis*. Two samples were found positive. Three passages were examined for colour change, at each passage the colour of the medium turned pink, and samples were thus considered as positive cultures. Proteinase K treated culture materials were analyzed by *Mycoplasma*-genus-specific PCR [35], which gives PCR products from 16S rRNA gene of 265 bp in size. The PCR products from the two positive clinical samples were sequenced and the resulting DNA sequence confirmed them to be *M. hominis*.

Quantification by culture of these two positive samples was performed by limited dilution in growth medium. There was a colour change in 9 wells in both samples, which corresponds to 25.600 CCU/ml in the swab sample, calculated from titration.

#### LightCycler PCR on DNeasy treated samples

**Figure 8:**
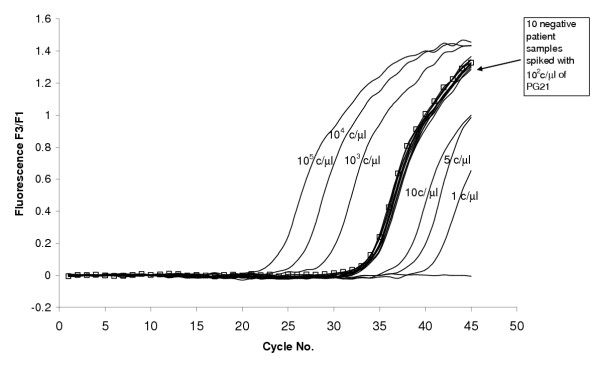
**Reproducibility of the LightCycler assay in clinical samples. **The LightCycler PCR run of 10 negative clinical samples spiked with the known PG21 DNA concentration of 10^2 ^copies/μl. The assay showed no inhibition, all curves came up at the same time as 10^2 ^copies/μl of PG21 (marked with squares) from the standard dilution series.

The cervical swab samples were DNeasy treated and tested in duplicates by LightCycler PCR using the *M. hominis gap*-assay. Two samples (nos. 56 and 83) were positive when examined by LightCycler PCR (2.4%). The copy numbers were measured to be 220 (for 83) and 530 (for 56) copies/μl respectively. The amount of DNA copies per ml in the original sample was calculated to be 37.000 for sample 83 and 88.500 for sample 56. The two quantification methods for estimating bacterial load in positive samples thus showed that the number of live *Mycoplasma *cells was 69% (patient 83) and 29% (patient 56) of what was found by PCR. The positive clinical samples showed melting temperature of 64°C corresponding to the second group, in which the majority of the *M. hominis *isolates were found. Reproducibility of the quantification of DNA in the clinical specimens was analyzed in 10 negative patient samples. Four μl of samples were spiked with 2 μl of PG21 DNA of known concentration (100 copies/μl). The six μl were added to the LightCycler PCR reaction. No inhibition was observed (fig. 8).

**Figure 9:**
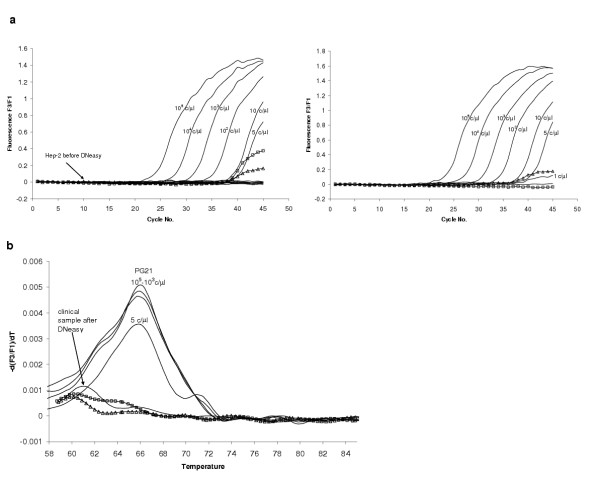
**DNeasy treated samples. **(a) Two LightCycler PCR runs, first one with standard dilution series of human Hep2 DNA before (flat negative curves) and after DNeasy (indicated with squares and triangles), showed on the left and second with DNA free water before (marked with squares) and after DNeasy (triangles) on the right. (b) Melting curve analysis of DNeasy treated H_2_O (triangles), Hep2 DNA (squares) and clinical sample.

In some DNeasy treated clinical samples a slight fluorescence response was seen at the very late PCR cycle. To analyze whether this slight fluorescence response was unspecific, different DNeasy samples were analyzed. We used different concentrations of human DNA from "*Mycoplasma *free" Hep2 cells, which were purified with Blood & Cell Culture DNA Mini Kit, and these samples did not give a positive fluorescence signal when run in LightCycler PCR (fig. 9a). In addition the results showed that there was no cross-reaction to human DNA. However, when DNA free water and standard dilutions of the human DNA extracted by the Blood & Cell Culture DNA Mini Kit were treated with DNeasy Tissue Kit, we experienced a slight fluorescence signal at the very late cycle numbers. Even DNA free water gave a positive signal (fig. 9a). The calculated copy numbers were between 1–8 copies/μl. The melting curve analysis showed an atypical flat and broad melting curve with the melting peak below the range of *Mycoplasma hominis *isolates (fig. 9b).

Clinical samples showing the low concentration (between 1–8 copies/μl) also had the flat melting curve with the melting peak below 61°C (fig. 9b) and were therefore considered as negative. One additional sample (no. 9) had an average concentration of 10 copies/μl, but when comparing the melting curve data, it had a melting curve identical to the DNeasy treated water and was therefore considered negative. There was thus 100% agreement between cultivation and detection by real-time PCR.

#### LightCycler PCR on proteinase K treated clinical samples

To additionally confirm that samples which gave a slightly positive fluorescence signal in LightCycler PCR after DNeasy treatment were not containing *M. hominis *DNA, we analysed proteinase K treated samples from those patients by the LightCycler PCR. None of the samples gave a positive fluorescence signal. After confirming that all samples were negative, we spiked 2 μl of each sample with 2 μl of PG21 DNA of 100 copies/μl, as the control for inhibition. None of the proteinase K treated samples showed inhibition. This clearly indicates that those samples were negative.

## Discussion

A rapid quantitative real-time PCR for detection of *M. hominis *from cervical swab samples was developed. To our knowledge it is the first LightCycler PCR protocol where quantification of *M. hominis *is combined with melting curve assay. The LightCycler PCR reproducibility was able to detect down to ten copies/reaction of the genomic PG21 DNA. All other 20 *M. hominis *isolates were positive in the assay. Additionally, isolates 132 and 4712, which based on melting temperature belong to the two other groups than PG21, were used to document detection limits of *M. hominis *isolates. Similarly to PG21, the detection limits were 5 copies/μl, equals to 10 copies per reaction for both isolates.

The average efficiency was high, but small differences were seen. This can be caused by different factors such as presence of inhibitors in the sample and treatment of the sample.

The artificially constructed samples consisting of PG21 DNA and coloured BEa and SP-4 media showed that these media inhibited the PCR reaction indicating that it was crucial to wash the pellets of other *Mycoplasma *species with PBS. Additionally, a spiking assay where DNA from other mycoplasmas was used together with DNA from PG21 showed that there was no inhibition in the proteinase K treated samples.

Since swab samples may contain PCR inhibitors we introduced the DNeasy procedure to additionally purify DNA from proteinase K treated clinical samples. Analysis of the ten negative clinical samples spiked with DNA from PG21 (100 copies/μl) showed no inhibition after DNeasy treatment.

We experienced, however, some slight fluorescence response in the very late cycle number, which corresponds to the low concentration seen in DNeasy treated water or Hep2 cells (fig. 9a) and flat melting curves with lower melting temperature than those of *M. hominis *isolates (fig. 9b). DNeasy treated patient samples that gave such a weak fluorescence signal were considered as negative. Additionally, using the original proteinase K treated patient samples, for those patients that gave a weak fluorescence signal after DNeasy treatment, we did not see any reaction with LightCycler PCR. This strongly indicates that such slight fluorescence response is generated only in the DNeasy treated samples.

A previous study showed that women with bacterial vaginosis showed presence of *M. hominis *in 38.5% compared to 8.3% of women with normal microbial flora [36]. Since none of our patients had bacterial vaginosis we had expected approximately 8% of our samples to be positive. We found two positive samples that were confirmed to be positive by the culture and had a high concentration of DNA by LightCycler PCR (220 and 530 copies/μl). The low number of positive patients (2.4%) was surprising but comparable with a previous study made on patients undergoing the *in-vitro *fertilization [7].

Comparison of the number of DNA copies and CCU showed that the CCU was lower than the number of DNA copies. This can be explained by the presence of dead bacteria in the samples.

Even though the assay was designed for quantification, the variability in the melting peaks gave additional information of *M. hominis*. Differences in the melting temperatures between *M. hominis *isolates prove heterogeneity of the housekeeping gene sequence. The *Tm *value determines how well the sequence of probes matches the sequence of template DNA, and it will decrease if mismatched DNA is amplified. Single mismatch can decrease the *Tm *from 1°C up to 30°C [37,38] depending on many factors, such as pH, duplex length and G + C content. This kind of analysis is used in detection of subtypes of Herpes simplex virus, since the *Tm *discriminates between two different subtypes [33,39,40]. In the present study, *Tm *of the clinical samples can suggest to which group of isolates they belong and how different they are from the PG21 template DNA. In this study, patient samples nos. 56 and 83 had melting peaks similar to isolates from the second and largest group.

The real-time technology where measurement of the fluorescence emitted during amplicon production is performed during each PCR cycle is considered as a breakthrough in PCR. Conventional PCR is an open, contamination-susceptible system where it is necessary to transfer the amplified product to other detection systems to confirm a positive result. Real-time PCR benefits by a closed system in which formation of a product is measured immediately without transfer [32]. Interpretation of LightCycler PCR results, presented as graphs and calculation of crossing points, introduces many advantages, but such a parameter of real-time PCR should be evaluated.

## Conclusions

LightCycler PCR appears very promising for detection of organisms that are difficult to culture or whose growth is slow. We have developed a quantitative, specific LightCycler protocol for detection of *M. hominis, *which offers rapid diagnosis of one hour after DNA extraction. The DNA extraction method used was not the best choice as unspecific fluorescence did occur in the late number of cycles. Results from cultivation and LightCycler PCR were identical. The method is both sensitive and specific. All tested isolates gave a positive fluorescence response, and final amplification and quantification was performed in closed tubes, which reduces the risk of contamination. The described target *gap *gene sequence should be preferred to more varying parts. A small variation in this part of the *gap *gene among different *M. hominis *isolates was observed by the melting curve analysis.

## Methods

### Microorganisms and human DNA used for the study

Organisms used in this study are listed in table 1.

### Subjects

A total of 83 consecutive women attending fertility clinics in Denmark (Brædstrup/Horsens and Holstebro) were studied. All patients were undergoing hysteroscopy and transvaginal hydrolaparoscopy (culdoscopy) [41] or laparoscopy due to infertility. The endocervical specimens for detection of presence of *M. hominis *were collected before the scopic examination.

### Material collection

Endocervical specimens were obtained using a sterile chlamydial swab and the contents transferred immediately into a tube containing 2 ml of transport medium, SP-4 [42], containing thallium acetate (0.01%), which inhibits growth of other microorganisms. Such prepared samples were then sent to the laboratory of Department of Medical Microbiology and Immunology, Aarhus University, where they were stored at -70°C.

### Cultivation and harvesting of microorganisms and human Hep2 cells used in the study

All isolates of *M. hominis, M. buccale, M. salivarium, M. orale, M. arthritidis, M. arginini, M. lipophilum, M. primatum *were cultivated in 1.7 ml of broth BEa medium [22]. *U. urealyticum *and *U. parvum *were grown in 1.7 ml of SU medium [43]. *M. pneumoniae *and *M. genitalium *were grown in 100 ml of SP-4 medium [42] as described in detail elsewhere [44]. *M. fermentans, M. pulmonis, M. hyorhinis *were cultivated in 1.7 ml of BEg medium [43]. Finally, *M. bovis *was cultured in BE medium [43]. All cultures were incubated at 37°C. The BEa medium changed colour from orange to pink in 48 hours due to reduction of phenol red by arginine hydrolysis. SP-4 and BEg both changed colour from orange to yellow, whereas SU changed from yellow to orange. BE medium changed from yellow to light pink in 72 hours. Except for *M. hominis, M. genitalium *and *M. pneumoniae*, which were cultivated up to bigger volumes (100 ml), 500 μl of the colour changed cultures were then placed in 7 ml of the new medium, and harvested after the medium changed for the second time. One ml of logarithmic-phase culture was centrifuged in Eppendorf tubes at 20.000 × g for 30 min. Each pellet was washed twice by phosphate-buffered saline (PBS) and the pellets were stored at -70° prior to use.

Human Hep2 cells were cultured as described elsewhere [45].

### DNA extraction and purification from microorganisms and human Hep2 cells used in the study

DNA from *M. arginini, M. bovis, M. hyorhinis, M. pulmonis, M. primatum*, *M. lipophilum, M. buccale, M. salivarium, M. orale, M. arthritidis, U. urealyticum, U. parvum, M. pneumoniae *and *M. fermentans *was extracted by suspending pellets in 160 μl TE buffer, and adding 40 μl 10 mg/ml proteinase K. The proteins were digested by incubation at 55°C for one hour, followed by inactivation of the enzyme by boiling for 10 minutes at 100°C.

The DNA from human Hep2 cells as well as *M. genitalium *DNA was extracted and purified by Blood & Cell Culture DNA Mini Kit (QIAGEN GmbH, Hilden, Germany).

Genomic DNA from all *M. hominis *isolates was extracted as described [46] and followed by ultracentrifugation in CsCl-ethidium bromide density gradient [47].

Concentrations of DNA from isolates PG21, 132 and 4712, and human Hep2 DNA were calculated after measuring OD (optical density) at 260 and 280 nm with Unicam 8625 UV/VIS spectrometer (ATI Unicam, Cambridge, United Kingdom).

### Artificial "Mycoplasma free" samples treated with DNeasy Purification Kit

Fourfold dilution series of human Hep2 DNA purified by Blood & Cell Culture DNA Mini Kit (QIAGEN GmbH, Hilden, Germany) of initial concentration 8.000 copies/μl were prepared. Twenty-five μl of each dilution and DNA free double distilled water were then treated with DNeasy™ Tissue Kit (QIAGEN GmbH, Hilden, Germany) procedure. Samples were diluted twice, because of elution step with 50 μl of elute buffer, and therefore 4 μl instead of two of each sample were used in the LightCycler PCR run.

### Cultivation of patient cervical samples

Broth BEa medium was used for culture of *M. hominis*. To avoid overgrowth by other bacteria, present in the urogenital tract, a special mixture of antibiotics (Niels Friis; containing: 0.15 mg/ml cycloserine, 0.2 mg/ml vancomycin, 0.2 mg/ml bacitracin and 0.2 mg/ml mecillinam) was used. Twenty μl of the swab sample was placed in 1 ml of medium with Niels Friis antibiotics and incubated at 37°C. The BEa medium changed colour from orange to strawberry pink in 48 hours due to reduction of phenol red by arginine hydrolysis. Finally, samples were described as positive when it was possible to pass them further 2 times. *Mycoplasma*-genus-specific PCR was performed on the two positive cultures and the PCR products were sequenced. Sequencing reactions were carried out bidirectionally using the ABI PRISM Dye Terminator Cycle Sequencing Ready Reaction Kit (Perkin Elmer, Narwalk, USA) on the purified PCR products according to the instructions supplied by the manufacturer. Sequencing was performed on an ABI PRISM 377 DNA Sequencer (Perkin Elmer, Narwalk, USA).

### DNA extraction from cervical samples

All 83 patient samples were treated identically. Briefly, three hundred μl of the original swab sample was subjected to microcentrifugation at 20.000 × *g*, and to remove transportation medium the pellets were washed twice with PBS. The pellets were suspended in 49 μl TE buffer, and 1 μl 10 mg/ml Proteinase K was added, proteins were digested while incubating at 55°C for one hour, which was followed by inactivation of the enzyme by boiling for 10 minutes at 100°C. Finally, in order to remove possible inhibitors present in swab samples, DNA extraction with DNeasy™ Tissue Kit (QIAGEN GmbH, Hilden, Germany) was performed on the resulting solution without repeating the proteinase K treatment. Twenty-five μl of the proteinase K treated sample was diluted twice because of elution with 50 μl of elute buffer, 4 μl of each sample was used for the PCR. The filter pipette tips were used for all DNA preparation steps to reduce possibility of sample contamination.

### Quantification of the number of viable microorganisms by culture

Estimation of the number of *M. hominis *in patient swab samples was performed by titration in BEa growth medium. Two-fold dilutions were made in ELISA trays by adding 0.01 ml of the clinical sample to 0.19 ml of BEa medium. The plates were incubated at 37°C and reading was performed on the third day. Plates were left in incubator and observed for 3 additional days but no further change appeared. The last well with visible colour change was considered to contain one colour changing unit (CCU) allowing us to calculate the number of viable microorganisms in the original clinical sample.

### Primer and probe design

Primers and probes were designed from the *gap *gene of *Mycoplasma hominis *type strain PG21 (Accession No. AJ243692). This gene belongs to the housekeeping genes and is therefore very conservative in all organisms. Primer and probe sequences and their locations are present in table 2. Both primers and probes were placed in front of the conserved region of the *gap *gene that is almost identical in all organisms. Primers were obtained from DNA Technology, Aarhus, Denmark, and probes from TIB-MOLBIOL, Berlin, Germany. Probe and primer sequences were analysed by BIOBASE (The Danish Biotechnological Database, the University of Aarhus, Denmark) and BLAST (National Centre of Biotechnology Information, National Institutes of Health, Bethesda, MD, USA).

### Real-time PCR assay with hybridization probes

The PCR product was 144 bp in size, which according to the manual (Roche Molecular Biochemicals Technical Note No. LC 11/2000) is preferable to perform an efficient quantification of DNA.

Real-time PCR was performed in glass capillary tubes. The reaction mixture was composed of 0.5 μM of each primer, 0.2 μM of each probe, 5 mM of MgCl_2 _(PCR buffer), 2 μl of ready-to-use Fast Start DNA Master Hybridization Probes (Roche Diagnostics, Mannheim, Germany) (contains a hotstart Taq DNA polymerase and reaction mixture), 1.5 μl Uracil-DNA Glycosylase (heat-labile) and 2 μl of the DNA template. Water was added up to 20 μl, which was the final volume of all reaction-mix. For the DNeasy treated patient samples 4 μl of DNA was used. When proteinase K treated patient samples were examined (without DNeasy treatment), we used 2 μl of the undiluted DNA.

To avoid contamination, mixing of the reagents (except of the DNA template) was performed in a separate room, away from rooms where culturing and DNA purification were done. The DNA template from *M. hominis *PG21 was added by use of filter pipette tips. Uracil-DNA Glycosylase (Roche Diagnostics, Mannheim, Germany) was used to prevent the samples from possible PCR "carry-over" contaminations from previous DNA synthesis reactions. Reaction mixes contained dUTPs instead of dTTPs, and therefore it was possible to avoid contamination of samples by adding an enzyme that hydrolyzes uracil-glycosidic bonds at U-DNA in single and double-strained DNA [48].

As negative control a sample containing all reagents except DNA was used in every PCR run. Quantification of DNA concentrations performed by LC-PCR Software was based on standard dilution series with known concentrations of genomic *M. hominis *PG21 DNA. The concentrations of standard dilution series were: 10^5^, 10^4^, 10^3^, 10^2^, 10^1^, 5 and 1 copy/μl. Siliconized tubes were used to prevent DNA from sticking to the wall of the plastic tubes. For carrier, yeast RNA was used in concentration 10 μg/ml.

The LightCycler PCR program was composed by: Hotstart Taq DNA polymerase activation done in 95°C for 10 minutes, followed by cycling: 95°C (20°C/s) for 15 s, 58°C (20°C/s) for 8 s and 72°C (20°C/s) for 8 s, repeated 45 times. Melting assay ended the analysis: samples were heated to 95°C (20°C/s) without hold, cooled to 55°C (20°C/s) hold for 15 s and then heated slowly at 0.1°C/s up to 95°C, finally cooled to 40°C (20°C/s).

Fluorescence emitted at 705 nm was measured at each annealing step since the fluorescence signal is emitted when both probes are hybridized. After annealing the temperature is raised and the hybridization probes are displaced by the Taq polymerase during the elongation step. Probe fluorescence was detected in canal F3 (measures at 705 nm) and F1 (measures at 530 nm). F3/F1 was used to correct differences in volume of the samples made during pipetting.

### Quantification of LightCycler products

The results were interpreted with LightCycler software Vers. 3.5 (Roche Diagnostics). Quantification software performs all additional steps for generation of a standard curve. First step involves *Baseline Adjustment *with the use of a "fit points" method, second step allows background reduction using *Noise Band *correction, and the last step is *Analysis *where the standard curve is generated from the threshold cycles (*C*_*t*_) of the standard dilution series (fig. 1b). Samples with high DNA load had low *C*_*t *_values, and low DNA load had high *C*_*t *_values. The concentration of DNA in clinical samples was set as "unknown". Each sample was run in duplicate. Calculation of the DNA concentration in the unknown sample was based on the standard curve slope. The average of the two concentration measurements was used for further analysis.

## Competing interests

None declared.

## Authors' contributions

Author A.B. carried out the real-time PCR experiments, the analyses of data, and drafted the manuscript. Author H.F.S. participated in designing the LightCycler PCR method, analysis of data and coordination of the manuscript. Author J.F. participated in coordination of the study and provided clinical samples. Author S.B. participated in design and coordination of the study. Author G.C. participated in design, data analyses, coordination of the manuscript and study.

## Figures and Tables

**Table 1:** List of the source of microbial and human DNA used in the assay

***M. hominis *isolates**	Isolation reference	Isolation Source	Accession no.*gap *gene
PG21	Nicol and Edward [20]	Lower genitalia	AJ243692
183	Linn and Kass [21]	vagina	

7357	Christiansen and Andersen [22]	cervix	AJ298001
6188	Christiansen and Andersen [22]	cervix	
3849	Christiansen and Andersen [22]	cervix	
P2	Thomsen [23]	upper urinary system	AJ298004
93	Linn and Kass [20]	vagina	AJ279227
4712	Christiansen and Andersen [22]	cervix	AJ297999
5941	Christiansen and Andersen [22]	cervix	AJ298000
DC63	Taylor-Robbinson [24]	cavum oralis	AJ298003
1893	Christiansen and Andersen [22]	cervix	
V2785	Taylor Robbinson [24]	cavum oralis	AJ298006
3105	Christiansen and Andersen [22]	cervix	AJ243694
W2	Lee [25]	wound	
P7	Thomsen [23]	upper urinary system	
SC4	Hollingdale and Lemcke [26]	urethra, male	AJ298005
132	Linn and Kass [21]	vagina	AJ243693
P71	Thomsen [23]	upper urinary system	
7808	Christiansen and Andersen [22]	urethra, female	AJ298002
M1449	Friis [27]	blood	
10	Linn and Kass [21]	vagina	

***Mycoplasma species***	Source		
*M. arginini*	G230		
*M. lipophilum*	MaBy		
*M. primatum*	HRC 292		
*M. pulmonis*	ASH PB34		
*M. hyorhinis*	AMRC 108		
*M. bovis*	Donetta PG45		
*M. buccale*	20247		
*M. fermentans*	PG18		
*M. genitalium*	G37		
*M. pneumoniae*	FH		
*M. salivarium*	PG20		
*M. orale*	CH19299		
*M. arthritidis*	PG6		
*U. urealyticum*	Serovar 8 (T960)		
*U. parvum*	Serovar 6 (Pirillo)		

**Human**	Source:		
**Genomic DNA**	Hep2; ATCC		

**Table 2:** Primers and probes sets used in the study

Oligonucleotide	Nucleotide position	Sequence
Forward Primer	295–322	5'-GGAAGA-TATGTAACAAAAGAAGGTGCTG-3'
Reverse Primer I	411–438	5'-TTTATCTTCTGGCGTAATGATATCTTCG-3'
Reverse Primer II	430–457	5'- ATGAAGCGCCTGATAAAATTTTATCTTC-3'
Probe I	336–368	5'-AGCAGGTGCTAAAAAGGTGTTTATTACTGCTCC-FL-3'
Probe II	370–408	5'-LCred705-GCTAAAAGCGAAGGTGTTAAAACAGTT GTTTATTCAGTA-3'

**Table 3:** Reproducibility of the LightCycler-PCR

Copy number	No. of PCR runs where fluorescence gave positive signal	CP	SD	CV % of CP
10^5^	10 / 10	25.4	1.7	6.7
10^4^	10 / 10	28.7	1.6	5.5
10^3^	10 / 10	32.3	1.6	4.8
10^2^	10 / 10	35.8	1.7	4.8
10^1^	10 / 10	39.7	2.1	5.3
5 copies	10 / 10	40.6	2.3	5.6
1 copy	6 / 10	43.8	1.7	3.8

CP = crossing-points, mean value

CV = coefficient of variation

SD = standard deviation

Statistical analysis of standard dilution series of standard PG21 DNA was performed. Ten different runs were analysed for crossing-points values and for presence of fluorescence signal. Based on crossing-points values the standard deviation of single concentration was calculated and coefficient of variation as a function: CV% = (SD / Average CP value for each concentration) × 100%.
